# Chemical composition, antioxidant and anticholinesterase potentials of essential oil of *Rumex hastatus* D. Don collected from the North West of Pakistan

**DOI:** 10.1186/s12906-016-0998-z

**Published:** 2016-01-25

**Authors:** Sajjad Ahmad, Farhat Ullah, Abdul Sadiq, Muhammad Ayaz, Muhammad Imran, Imdad Ali, Anwar Zeb, Farman Ullah, Muhammad Raza Shah

**Affiliations:** 1 Department of Pharmacy, University of Malakand, Chakdara 18000, Dir (L), KPK Pakistan; 2 International Center for Chemical and Biological Sciences, H.E.J. Research Institute of Chemistry University of Karachi, Karachi, Pakistan; 3 Department of Pharmacy, Kohat University of Science &Technology, Kohat, Pakistan

**Keywords:** Essential oil, Acetylcholinesterase, Butyrylcholinesterase, Antioxidant, GC-MS, Free radicals, *Rumex hastatus*

## Abstract

**Background:**

Ethnomedicinally *Rumex hastatus* D. Don has been used since long for various ailments especially in neurological disorders. The reported data and the importance of Rumex genus demonstrate the vital medicinal value of *R. hastatus*.

**Methods:**

In the current investigational study, isolation of essential oil and its antioxidant and anticholinesterase assays were performed. The essential oil of *R. hastatus* was analyzed by GC-MS for the first time. The essential oil was evaluated for anticholinesterase and antioxidant assays. The anticholinesterase assay was conducted at various concentrations (62.5 to 1000 μg/ml) against acetylcholinesterase (AChE) and butyrylcholinesterase (BChE). Similarly, the antioxidant potential was determined using DPPH and ABTS free radicals.

**Results:**

The GC-MS analysis of essential oil showed 123 components. The result recorded for the anticholinesterase assays demonstrated a marked potential against AChE and BChE with IC_50_ values of 32.54 and 97.38 μg/ml respectively which were comparable with the positive control i.e., galanthamine (AChE, IC_50_ = 4.73 μg/ml and BChE, IC_50_ = 11.09 μg/ml). The antioxidant assays against DPPH and ABTS free radicals also exhibited significant scavenging potential with IC_50_ values of 3.71 and 6.29 μg/ml respectively, while for ascorbic acid the IC_50_ value was <0.1 μg/ml against both free radicals.

**Conclusions:**

Based on the current investigational studies, it may be concluded that *R. hastatus* is an effective source of essential oil's components having anticholinesterase and antioxidant potentials, which after subjecting to drug development may lead to novel drug candidates against neurodegenerative disorders.

## Background

A brief history of medicine demonstrates the use of herbal medicine for the effective treatment of various ailments. Herbal medicine has been used since long in various forms including the decoction, powdered sample, oleoresins, crude extracts, fixed oil, essential oil etc [1]. Various plants have been used in multiple types of food items for preservation and therapeutic effects [2]. In this regards, essential oils have been manifested by several reporters to play a major role. Essential oils have the property to attenuate the effects of free radicals, e.g, reactive oxygen species (ROS) which are derived from metabolism of oxygen and exogenous agents [3]. ROS are responsible for wide variety of diseased conditions including oxidative stress and nervous disorders [4]. Essential oils are well-known for their radicals scavenging properties and amelioration of various cognitive disorders. Among the cognitive disorders, Alzheimer’s disease (AD) is the most common in elderly people [5]. One of the best therapeutic approaches for AD is to increase the concentration of the neurotransmitter (Acetylcholine) by inhibiting the enzyme (acetylcholinesterase) responsible for its breakdown. Various drugs originated either from natural or synthetic sources are being used for the management of AD and other nervous disorders [6]. Similarly, it has also been reported that oxidative stress are responsible for wide variety of mental diseases due to neuronal degeneration and other factors. Oxidative stress is mainly developed due to increase in concentration of free radicals within the body. The free radicals have been reported by numerous researchers to possess multiple destructive properties, due to which interest has been focused to scavenge the free radicals somehow and avoid their deteriorating effects [7]. In this context, investigators are trying to explore more and more sources of natural and synthetic bioactive principles [8]. The natural drugs are being preferred over the synthetic due to their negligible harmful and deleterious effects [9]. That’s why researchers are trying to explore novel sources of natural medicine [10–18]. Among the natural sources, herbal medicines have been shown promising results due to the presence of numerous secondary metabolites and essential oils. Essential oils isolated from various plants have been reported to possess marked acetylcholinesterase inhibitory and radicals scavenging potential [19–21]. Traditional knowledge also demonstrates the use of essential oils for various nervous system disorders [22].

*R. hastatus* D. Don belongs to the family Polygonaceae. Various members of this family have been reported to be used against paralysis, headache and other nervous system disorders [23–26]. Various solvent samples of *R. hastatus* have recently been reported to possess strong anticholinesterase and antioxidant potentials [26]. To date, the chemical composition of essential oil of *R. hastatus* has not been reported or evaluated for any pharmacological activity. Based on the literature survey and medicinal importance of *R. hastatus*, the current investigational study is arranged to isolate the essential oil, analyze the chemical composition and to evaluate for the anticholinesterase and antioxidant potentials, which may be a possible remedy for oxidative stress and nervous system disorder.

## Methods

### Plant sample collection

The aerial parts of *R. hastatus* were collected from the proximity of University of Malakand. The plant was identified by plant taxonomist Ali Hazrat and deposited with voucher number (1015SJ) in the herbarium of Department of Botany, Shaheed Benazir Bhutto University Sheringal, Dir (U), KPK, Pakistan. Extraction of essential oil of *R. hastatus* was performed by hydrodistillation using clevenger type apparatus [27]. The essential oil obtained was stored at -20 °C until required.

### Chemicals and drugs

DPPH (Sigma Aldrich CHEMIE GmbH USA, code 101341986), K_2_S_2_O_4_ (Riedel-de Haen Germany), ABTS (Sigma Aldrich USA, code 1001551916), Gallic acid (GmbH USA), Folin Ciocalteu reagent (Merck Co. Germany). AChE (Electric eel type-VI-S, Sigma-Aldrich GmbH USA, code 1001596210), BChE (Equine serum Lyophilized Sigma-Aldrich GmbH USA, code 101292670), Acetylthiocholine iodide (Sigma-Aldrich UK, code 101303874), Butyrylthiocholine Iodide (Sigma-Aldrich Switzerland, code 101334643), DTNB (Sigma-Aldrich Germany, code 101261619), Galanthamine hydrobromide Lycoris Sp. (Sigma-Aldrich France, code G1660). K_2_HPO_4_, KH_2_PO_4_, KOH. All the chemical used were of analytical grade.

### Gas Chromatography (GC) analysis

The GC analysis of essential oil was carried out via gas chromatograph Agilent USB-393752 (Agilent Technologies, Palo Alto, CA, USA) with HHP-5MS 5 % phenylmethyl siloxane capillary column (30 m × 0.25 mm × 0.25 μm film thickness; Restek, Bellefonte, PA) connected with FID detector. The oven was set at temperature of 70 °C for one minute and then increased to 180 °C at the rate of 6 °C/min for 5 min and lastly to 280 °C at the rate of 5 °C/min for 20 min. The temperature of injector and detector were maintained at 220 °C and 290 °C correspondingly. The flow rate of carrier gas i.e., Helium was 1 ml/min and the diluted samples (1/1000 in *n*-pentane, v/v) of 1 μl were manually injected in the split-less mode.

### Gas Chromatography–Mass Spectrometry (GC-MS) analysis

The GC/MS of the essential oil was performed via USB-393752 gas chromatograph (Agilent Technologies, Palo Alto, CA, USA) with a HHP-5MS 5 % phenylmethyl siloxane capillary column (30 m × 0.25 mm × 0.25 μm film thickness; Restek, Bellefonte, PA) outfitted with an Agilent HP-5973 mass selective detector in the electron impact mode (Ionization energy: 70 eV) working under the experimental conditions as those maintained for GC.

### Identification of components

The recognition of all the major constituents of oil was performed by comparing their retention times with the authentic compounds in the literature. Identification of compounds was further processed through the spectral data obtained from the Wiley and NIST libraries as well as fragmentation patterns’ comparisons of the mass spectra with data reported in literature or with those of mass spectra from literature [28, 29]. Each determination was processed in duplicate.

### Anticholinesterase assays

Anticholinesterase (AChE and BChE inhibitions) activity was performed for the essential oil of *R. hastatus* by spectrophotometric analysis following the method of Ellman's assay [30]. The substrates used were acetylthiocholine iodide and butyrylthiocholine iodide. Briefly, 5 μL of 0.03 U/mL AChE and 0.01 U/mL BChE were taken in a cuvette and 205 μL of essential oil having concentration of 62.5–1000 μg/mL were transferred to them using micropipette. Similarly, 5 μLof DTNB was also added to this afterwards. The mixtures obtained were kept in water bath for 15 min at the temperature of 30 °C. After incubation, 5 μL of the Substrates were added to the mixture to optimize the reaction. A double beam spectrophotometer was used to measure the reaction time at 412 nm via a double beam spectrophotometer (Thermo electron corporation USA). Absorption values were obtained for 4 min. Meanwhile, the yellow colored mixtures indicated the formation of 5-thio-2-nitrobenzoate anion as a reaction product of thiocholines and DTNB. White assay was also performed without enzymes and plant samples to check the non-enzymatic hydrolysis of substrate. The mixture which contained all the components excluding essential oil was marked as control. Percent enzyme activity and percent inhibition were recorded as follows.

$$ \mathrm{V} = \frac{\varDelta \mathrm{Abs}}{\varDelta \mathrm{t}} $$

$$ \%\ \mathrm{enzyme}\ \mathrm{activity} = \frac{\mathrm{V}}{{\mathrm{V}}_{\max }}\times 100 $$

$$ \%\ \mathrm{enzyme}\ \mathrm{inhibition}=100-\%\ \mathrm{enzyme}\ \mathrm{activity} $$

(Where V symbolizes the rate of reaction in the presence of inhibitor and V_max_ stands for rate of reaction without inhibitor)

### DPPH radical scavenging assay

The DPPH radical scavenging potential was evaluated for essential oil of *R. hastatus* following previously described procedure [31]. DPPH solution (0.004 %) was prepared in methanol to get a deep violet colored solution. Similarly, stock solution of essential oil was prepared in ethanol having concentration of 1 mg/mL. The stock solution was serially diluted to get the concentrations of 62.5 to 1000 μg/mL. Afterwards, 0.1 mL of each concentration was added to the 3 mL of DPPH solution. The mixture obtained was incubated at 23 °C for 30 min in dark. After incubation the absorbance of each sample were recorded at the wavelength of 517 nm using double beam spectrophotometer. Ascorbic acid was used as positive control. All the samples were processed in triplicates and the percent activity was recorded as mean ± SEM. The percent radical scavenging potential was figured out using the following formula;

$$ \%\ \mathrm{scavenging}=\frac{\mathrm{absorption}\ \mathrm{of}\ \mathrm{control}-\mathrm{absorption}\ \mathrm{of}\ \mathrm{test}\ \mathrm{sample}}{\mathrm{absorption}\ \mathrm{of}\ \mathrm{control}}\times 100 $$

### ABTS radical scavenging assay

The 2, 2-azinobis [3-ethylbenzthiazoline]-6-sulfonic acid (ABTS) free radicals scavenging assay of the essential oil was evaluated followed standard procedure [11]. ABTS solution 7 mM and potassium persulfate solution 2.45 mM were prepared and mixed thoroughly. The solution prepared was put in dark overnight for the production of free radicals. After incubation time the absorbance of solution was adjusted at 745 nm to 0.7 by the addition of 50 % methanol. Test samples having volume of 300 μl was taken in a test tube and 3 mL ABTS solution was added to it. The solution was transferred to the cuvette and absorbance values were taken for six minutes using double beam spectrophotometer. Ascorbic acid was used as positive control. All the samples were run in triplicate and percent ABTS radical scavenging potential was figured out using the following formula;

$$ \%\ \mathrm{scavenging}\ \mathrm{activity}=\frac{\mathrm{control}\ \mathrm{absorbance}-\mathrm{sample}\ \mathrm{absorbance}}{\mathrm{control}\ \mathrm{absorbance}}\times 100 $$

### Estimation of IC_50_ values

The median inhibitory concentration i.e., IC_50_ values of AChE, BChE, DPPH and ABTS were determined by a linear regression analysis of the percent inhibition versus the concentrations of test samples through MS Excel program.

### Statistical data analysis

All the tests were conducted in triplicate and the values were tabulated as mean ± S.E.M. Significant difference of the percent inhibition of various test samples was analyzed via two way ANOVA following Bonferroni’s post test using GraphPad Prism software in which the *P* < 0.05 were considered significant.

## Results and discussion

In the current investigational study the radical scavenging potential of volatile oil was studied based on spectrophotometric analysis. The sources of free radicals employed were DPPH and ABTS, which have maximum absorbance values at 517 nm and 745 nm respectively. After getting scavenged by antioxidant compounds the colors of DPPH (violet) and ABTS (blue) solution change into yellow. Change in the color results in decrease of absorbance values which is directly proportional to the amount of radical scavenging compounds in the solution [32, 33].

Similarly, the anticholinesterase activity is based on the hydrolysis of acetylthiocholine iodide and butyrylcholine iodide by the formation of the yellow 5-thio-2- nitrobenzoate anion as a result of the reaction of DTNB with thiocholines, catalyzed by enzymes at a wavelength of 412 nm using spectrophotometer or microplate reader. Acetylthiocholine iodide and butyrylthiocholine iodide work as substrate of the reaction, while the DTNB is utilized for the measurement of cholinesterase activity. The percent inhibition of enzymatic activity is calculated from the rate of change in absorption of the reaction mixture [34].

**Fig. 1:**
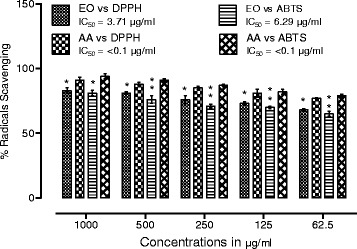
Antioxidant potential of essential of *Rumex hastatus* against DPPH and ABTS

**Fig. 2:**
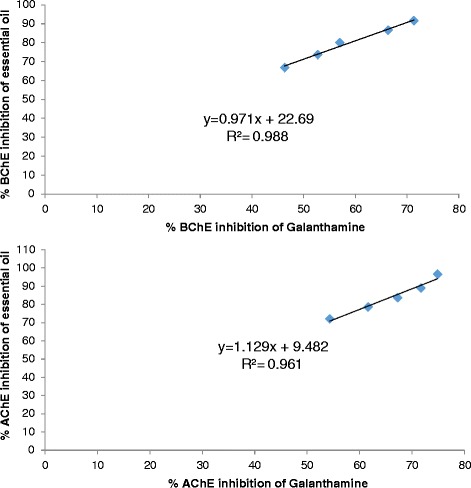
**a.** Regression and correlation of percent BChE inhibition of essential oil Vs Galanthamine. **b.** Regression and correlation of percent AChE inhibition of essential oil Vs Galanthamine

The available literature on etiology of diseases demonstrate multiple causative agents responsible for specific disease [35]. In the context of Alzheimer’s disease, numerous investigators have reported the role of various causative agents along with various successful approaches [36]. Like all neurodegenerative disorders, the free radicals have a prominent role in the induction and progression of AD [37]. By avoiding or attenuating the causative agents one can hinder the progression of a specific disease. In case of neurodegenerative disorders, the scavenging of free radicals can be a vital target. Various researchers have demonstrated the effective role of natural antioxidants especially the essential oils to combat the free radicals [38]. Similarly, one of the most widely employed treatment strategies for AD i.e., the inhibition of AChE to increase the concentration of neurotransmitter is highly recommended [39]. In this regard, essential oils are being investigated by advanced researchers with better results. Essential oils obtained from various plants possess marked anti-Alzheimer’s potential due to the presence of wide variety of valuable compounds in it [40, 41]. The anticholinesterase potential of essential oil of *Rumex hastatus* has been summarized in the Table 1, while the Table 2 shows various parameters of the compounds present in the essential oil of this plant. The GC-MS analysis of essential oil of *R. hastatus* demonstrates a total of 123 components as shown in Table 3. The anticholinesterase activity of essential oil of *R. hastatus* might be due to its hydrophobic nature because of the good affinity of hydrophobic active site of AChE [42, 43]. Some of the most common components of essential oils i.e., palmitic acid, myristic acid, pelargic acid, capric acid, docosane, cetane, velleral, acetone, methyl palmitate, widdrol, isolongifolol, ophytadiene, drimenol and levulinic acid have been found in the essential oil of *R. hastatus*. Some of these components have been reported previously by other investigators to possess antioxidant and anticholinesterase potentials [44–49]. The percent antioxidant potential of essential oil is illustrated in the Fig. 1. The peaks given in the Table 2 shows various volatile compounds like 5-ethyl-2(5H)-furanone, trimethylacetic anhydride, cyclooctanone, 5-methyl-3-heptanol, methyl 2-vinylbutanoate, 2-(*p*-methylphenyl)-2-nitropropne, azelaaldehydic acid, 2,4,6-trimethyloctane and *trans*-3-nonen-2-one with retention times of 6.447, 6.818, 10.958, 11.363, 11.761, 12.97, 13.171, 13.308, 15.063 and 19.213 min respectively. Going to the detail of various components of essential oil of *R. hastatus*, it is clear that the marked anticholinesterase potential shown by essential oil is observed due to the presence of wide variety of compounds in it. Essential oil demonstrated 74.90, 71.70, 67.26, 61.64, 54.32 % AChE inhibition at 1000, 500, 250, 125, 62.5 μg/ml respectively. Similarly, the BChE inhibition exhibited by essential oil was recorded as 71.32, 66.33, 46.32, 52.73, 57.00 % at 1000, 500, 250, 125, 62.5 μg/ml respectively. The essential oil attain IC_50_ values of 32.54 and 97.38 μg/ml for AChE and BChE inhibitions respectively. The anticholinesterase potential shown by essential oil goes parallel with the positive control which is also obvious from the Fig. 2 (a & b) with the correlation coefficient of 0.961 and 0.988 for essential oil versus AChE and BChE respectively. Apart from the anticholinesterase potential of essential oil, the antioxidant potential of essential oil of various plants has been reported with discrimination by various investigators [50, 51]. In our current investigational study, the free radicals scavenging assay of essential oil of *R. hastatus* against DPPH and ABTS was significant and almost comparable with the positive control. From Fig. 1, it is clear that essential oil exhibited marked potential with IC_50_ of 3.71 and 6.29 μg/ml against DPPH and ABTS respectively, which is also comparable with the previously reported literature. The previously reported data of *R. hastatus* verifies its anticholinesterase and antioxidant potentials which may be linked to the current investigational studies [26].

**Table 1:** Anticholinesterase activity of essential oil of *Rumex hastatus* at various concentrations

Samples	Enzymes	Conc. μg/ml	Conc. μg/ml	Conc. μg/ml	Conc. μg/ml	Conc. μg/ml	IC_50_ μg/ml
		62.5	125	250	500	1000	
EO	AChE	54.32 ± 1.33	61.64 ± 1.60	67.26 ± 1.24	71.70 ± 1.63	74.90 ± 0.52	32.54
EO	BChE	46.32 ± 3.50	52.73 ± 0.78	57.00 ± 2.80	66.33 ± 0.49	71.32 ± 4.8	97.38
Gal	AChE	72.08 ± 1.04	78.58 ± 1.12	83.70 ± 1.60	89.00 ± 1.15	96.65 ± 1.34	04.73
Gal	BChE	66.87 ± 1.27	73.67 ± 0.88	79.95 ± 2.01	86.62 ± 1.67	91.61 ± 0.43	11.09

Data is expressed as Mean ± SEM; EO and Gal are abbreviated for Essential oil and Galanthamine respectively

**Table 2:** Parameters of various components of essential oil of *Rumex hastatus*

RT (min)	Height	Height (%)	Area	Area (%)	Area Sum %	Base Peak m/z	Width
6.447	254413	18.51	620057	20.82	5.87	83	0.127
6.818	324110	23.59	626045	21.02	5.93	57.1	0.077
10.958	430958	31.36	822529	27.61	7.79	55.1	0.074
11.363	250143	18.2	592697	19.9	5.61	59.1	0.09
11.761	278058	20.23	665761	22.35	6.31	59.1	0.094
12.97	177060	12.88	399792	13.42	3.79	43.1	0.097
13.171	312841	22.77	664487	22.31	6.29	55.1	0.08
13.308	1E + 06	100	3E + 06	100	28.21	57.1	0.1
15.063	159790	11.63	336861	11.31	3.19	55.1	0.08
19.213	450356	32.77	782083	26.26	7.41	133.1	0.064

**Table 3:** List of components of essential oil of *Rumexhastatus*

S.No	Compound Label	Common name	RT	Formula	Hits (DB)
1.	Trans-dideuterioxy-cyclopentene	NF	5.757	C5H6D2O2	10
2.	1-Nonen-4-ol	NF	5.884	C9H18O	10
3.	Ethyl 2-hydroxybutyrate	NF	6.169	C6H12O3	10
4.	2(5H)-Furanone, 5-ethyl	NF	6.445	C6H8O2	10
5.	Pentanoic acid, 4-oxo	Levulinic acid	6.68	C5H8O3	10
6.	2,2-Dimethylpropanoic anhydride	Trimethylacetic anhydride	6.819	C10H18O3	10
7.	Heptanoic acid	Enanthic acid	7.117	C7H14O2	10
8.	Ethanethioic acid, S-(2-methylpropyl) ester	NF	7.374	C6H12OS	10
9.	4-Octanol, 7-methyl	NF	7.511	C9H20O	10
10.	4-(Tetrahydrofuranyl-2-oxy)-4-methyl-2-pentanone	NF	7.619	C10H18O3	10
11.	Cyclopropane, 1,2-dimethyl-1-pentyl	NF	7.698	C10H20	10
12.	n-Nonanal	Nonanal	7.852	C9H18O	10
13.	Cyclooctanone	NF	8.275	C8H14O	10
14.	1,4,4-Trimethylcyclohexa-2-en-1-ol	NF	8.494	C9H16O	10
15.	3-Octanol, 2-methyl	NF	8.716	C9H20O	10
16.	2-Oxatricyclo[3.3.1.1(3,7)]decane, 1-methyl-	NF	9.116	C10H16O	10
17.	Succinimide, N-methoxy	NF	9.338	C5H7NO3	10
18.	4-Heptanol, 2-methyl	NF	9.547	C8H18O	10
19.	Ethanone, 1-(methylphenyl)	Methylacetophenone	9.712	C9H10O	10
20.	Decanal	NF	10.099	C10H20O	10
21.	3-Heptanol, 2,4-dimethyl	NF	10.328	C9H20O	10
22.	Cyclooctanone	NF	10.957	C8H14O	10
23.	1-Decyne (CAS) $$ Octylacetylene	NF	11.165	C10H18	10
24.	3-Heptanol, 5-methyl	NF	11.364	C8H18O	10
25.	Nonanoic acid	Pelargic acid	11.456	C9H18O2	10
26.	ETHYL AMYL CARBINOL	NF	11.763	C8H18O	10
27.	CIS-SABINENE HYDRATE	NF	11.96	C10H18O	10
28.	1,8-Bisoxiranylnonane	NF	12.047	C13H24O2	10
29.	3-Heptanone, 4-methyl	NF	12.817	C8H16O	10
30.	Methyl 2-vinylbutanoate	NF	12.972	C7H12O2	10
31.	trans-3-Nonen-2-one	NF	13.171	C9H16O	10
32.	Octane, 2,4,6-trimethyl	NF	13.309	C11H24	10
33.	2H-Pyran-2-one, 6-heptyltetrahydro	Delta.-laurolactone	13.471	C12H22O2	10
34.	Decanoic acid	Capric acid	13.601	C10H20O2	10
35.	3-Octanol	NF	14.002	C10H22O	10
36.	Ethyl 3,3-dimethylbutyrate	NF	14.246	C8H16O2	1
37.	5-Hexenal	NF	14.547	C6H10O	10
38.	2-Pentenoic acid, 4-hydroxy	NF	14.878	C5H8O3	10
39.	Nonanoic acid, 9-oxo-, methyl ester	Azelaadehydic acid	15.065	C10H18O3	10
40.	Thiophene, 2-methoxy	NF	15.345	C5H6OS	3
41.	Octanoic acid, 8-hydroxy	NF	15.49	C8H16O3	10
42.	Oxirane, octyl	NF	15.604	C10H20O	10
43.	Butane, 1,1'-oxybis[3-methyl	NF	15.875	C10H22O	5
44.	3-Hydroxy-4-methoxystyrene	NF	16.153	C9H10O2	7
45.	Octanoic Acid	n-Caprylic acid	16.355	C8H16O2	10
46.	3-Hexanol, 3,5-dimethyl	NF	16.55	C8H18O	10
47.	2-Tridecen-1-ol, (E)	NF	16.643	C13H26O	10
48.	1-Isopropyl-4,7-dimethyl-1,2-dihydronaphthalene	Alpha-Calcorene	16.877	C15H20	10
49.	4-(5',5'-dimethyl-2'-methylidene-3',8'-dioxabicyclo[5.1.0]oct-4-ylidene)-2-b…	NF	17.084	C13H18O3	5
50.	9-Methyl-S-octahydrophenanathracene	NF	17.192	C15H20	10
51.	Z-10-Tetradecen-1-ol acetate	NF	17.373	C16H30O2	10
52.	Dodecanamide, N,N-bis(2-hydroxyethyl)	NF	17.737	C16H33NO3	10
53.	5,8-Dimethyl-1,2,3,4-tetrahydro-1-naphthol	NF	17.847	C12H16O	3
54.	3-Hexen-1-ol, benzoate, (Z)	NF	17.917	C13H16O2	10
55.	Nonanoic acid	Pelargic acid	18.014	C9H18O2	10
56.	Nonanedioic acid, monomethyl ester	NF	18.153	C10H18O4	10
57.	(-)-Caryophyllene oxide	Caryophyllene oxide	18.311	C15H24O	10
58.	(+-)-Andirolactone	Andirolactone	18.513	C11H14O2	10
59.	Ledol	NF	18.64	C15H26O	10
60.	(. + -.)-2-Methyl-6-p-tolyl-4-heptanol (diastereoisomer II)	NF	18.693	C15H24O	9
61.	Propanal, 2,2-dimethyl	NF	18.777	C5H10O	1
62.	2,6,10-Trimethylundecan-(5E)-2,5,9-trien-4-one	NF	18.869	C14H22O	10
63.	7-oxabicyclo[4.1.0]heptane, 1-(1,3-dimethyl-1,3-butadienyl)-2,2,6-trimethyl-	NF	19.004	C15H24O	10
64.	Octanoic acid, 6,6-dimethoxy-, methyl ester	NF	19.087	C11H22O4	10
65.	2-(p-methylphenyl)-2-nitropropane	NF	19.212	C10H13NO2	10
66.	Azelaic Acid	Anchoic acid	19.589	C9H16O4	4
67.	cis-9-oxabicyclo[6.1.0]non-2-ene	NF	19.736	C8H12O	10
68.	1-Buten-3-one, 1-(2-carboxy-4,4-dimethylcyclobutenyl)	NF	19.864	C11H14O3	10
69.	Campherenone	Campherenone	20.056	C15H24O	10
70.	11-Hexadecyn-1-ol	NF	20.231	C16H30O	10
71.	Cyclodecene, 1-ethyl-2-methyl-	NF	20.385	C13H24	10
72.	1,3-Dioxolane-4,5-dicarboxylic acid, 2,2-dimethyl-, dimethyl ester	NF	20.627	C9H14O6	5
73.	10-(1-Methylallyl)tricyclo[6.3.1.0(2,7)]dodeca-2(7),3,5-trien-10-ol	NF	20.768	C16H20O	4
74.	2-Acetoxy-1,1,10-trimethyl-6,9-epidioxydecalin	NF	20.894	C15H24O4	10
75.	Farnesyl Acetone C	Farnesyl Acetone	21.18	C18H30O	10
76.	17-Octadecynoic acid	NF	21.401	C18H32O2	10
77.	Tetradecanoic acid	Myristic acid	21.82	C14H28O2	10
78.	Driminol	Drimenol	22.167	C15H26O	10
79.	2,2,6-Trimethyl-1-(3-methylbuta-1,3-dienyl)-7-oxabicyclo[4.1.0]heptan-3-ol	NF	22.272	C14H22O2	10
80.	1,3,5-trimethyl-6-methyliden-tricyclo[3.2.1.0(2,7)]oct-3-en-8-endo-ol	NF	22.677	C12H16O	9
81.	1-Methyl-2-acetyl-6-methoxy-3,4-dihydronaphthalene	NF	22.933	C14H16O2	10
82.	N-(1-Cyanoethyl)(7,7-dimethyl-2-oxobicyclo[2.2.1]hept-1-ylmethanesulfonamide	NF	23.386	C13H20N2O3S	10
83.	5-(ethylamino)-1,6-dimethyl-2(1H)-quinolinone	NF	23.511	C13H16N2O	10
84.	(-)-Isolongifolol	Isolongifolol	23.926	C15H26O	10
85.	Neophytadiene	Neophytadiene	24.02	C20H38	10
86.	Naphthalene, 1-(1,1-dimethylethyl)-7-methoxy-	NF	24.123	C15H18O	2
87.	2-Pentadecanone, 6,10,14-trimethyl	NF	24.218	C18H36O	10
88.	2,5,8-Trimethyltricyclo[5.3.1.1(3,9)]dodecane-2-anti,8-tnti-diol	NF	24.561	C15H26O2	3
89.	Pentadecanoic acid	Pentadecyclic acid	24.74	C15H30O2	10
90.	9,19-Cycloergost-24(28)-en-3-ol, 4,14-dimethyl-, acetate	NF	25.047	C32H52O2	4
91.	8-Keto-10-dehydrobrominated-.beta.-snyderol	NF	25.298	C15H22O2	2
92.	Widdrol	Widdrol	25.848	C15H26O	10
93.	2,4,7,9-Tetramethyl-5-decyne-4,7-diol	NF	26.036	C14H26O2	4
94.	Phenol, 2-methyl-4-(1,1,3,3-tetramethylbutyl)	NF	26.54	C15H24O	10
95.	Benzene, 1,1'-(1,2-diethyl-1,2-ethanediyl)bis[4-methoxy-	NF	26.548	C20H26O2	10
96.	(1R,3S)-2,2,3-Trimethyl-6-methylidenecyclohexane-1-carbaldehyde	NF	26.624	C11H18O	5
97.	Hexadecanoic acid, methyl ester	Methyl palmitate	26.732	C17H34O2	10
98.	1-Hexadecen-3-ol, 3,5,11,15-tetramethyl-	NF	27.371	C20H40O	10
99.	Benzo[e]isobenzofuran-1,4-dione,1,3,4,5,5a,6,7,8,9,9a-decahydro-6,6,9a-trime…	NF	27.585	C15H20O3	10
100.	Hexadecanoic acid	Palmitic acid	27.984	C16H32O2	10
101.	Butane-1,1-dicarbonitrile, 1-cyclohexyl-3-methyl-	NF	28.431	C13H20N2	10
102.	2-Methyl-2-propyl-2,5-dihydrofuran	NF	28.552	C8H14O	10
103.	5A-Methyl-3,8-dimethylene-2-oxododecahydrooxireno[2',3':6,7]naphtho[1,2-b]fu…	NF	28.643	C20H24O5	10
104.	4-(3,7,7-Trimethyl-2-oxabicyclo[3.2.0]hept-3-en-1-yl)but-3-en-2-one	NF	28.98	C13H18O2	10
105.	Cyclobutanecarboxylic acid, 2-methyloct-5-yn-4-yl ester	NF	29.064	C14H22O2	10
106.	Cyclooctenone, dimer	NF	29.439	C16H24O2	10
107.	Undecane, 6-cyclohexyl-	NF	29.639	C17H34	10
108.	2,4,5,7-Tetramethyl-2,6-octadiene	NF	30.471	C12H22	10
109.	Cyclohexane, 1,2,3,4,5,6-hexaethyl	NF	30.77	C18H36	10
110.	Cyclopentanone, 3-methyl-2-(2-pentenyl)-	NF	31.291	C11H18O	10
111.	2-Propanon	Acetone	31.44	C3H6O	10
112.	beta.-Ionol $$ 3-Buten-2-ol, 4-(2,6,6-trimethyl-1-cyclohexen-1-yl)-	NF	31.703	C13H22O	10
113.	Velleral	Velleral	32.121	C15H20O2	10
114.	2-Hydrazino-2-imidazoline	NF	32.733	C3H8N4	10
115.	2H-cyclopropa[g]benzofuran, 4,5,5A,6,6A,6B-hexahydro-4,4,6b-trimethyl-2-(1-m…	NF	33.658	C15H22O	10
116.	Hexadecane	Cetane	37.132	C16H34	10
117.	Docosane	Docosane	38.808	C22H46	10
118.	4,4-6-Trimethyl-7-oxabicyclo[4.1.0]heptan-2-one	NF	39.247	C9H14O2	10
119.	1,2-Benzenedicarboxylic acid, bis(2-ethylhexyl) ester	NF	39.623	C24H38O4	10
120.	4-Allyl-1-ethoxy-3-phenylbenzo[c]-(1,2)-oxaphosphinine - 1-Oxide	NF	40.4	C19H19O3P	3
121.	Hexadecane	Cetane	41.915	C16H34	10
122.	Undecane, 3,8-dimethyl-	NF	44.76	C13H28	10
123.	4-Methyl-7-ethylizidine $$ 8-Methyl-5-ethylindolizidine	NF	58.237	C11H21N	10

**Fig. 3:**
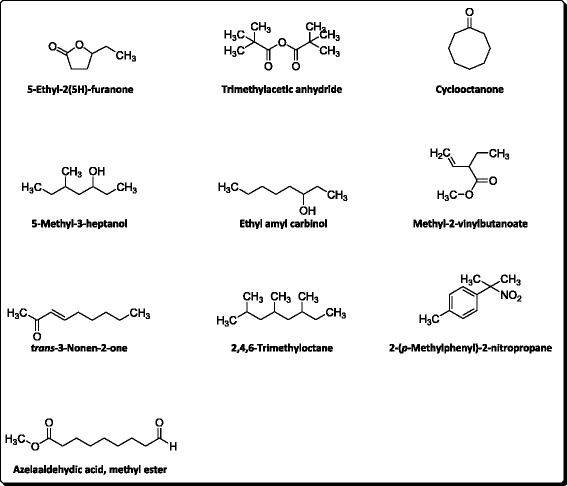
Structures of some important components of essential oil of *Rumex hastatus*

**Fig. 4:**
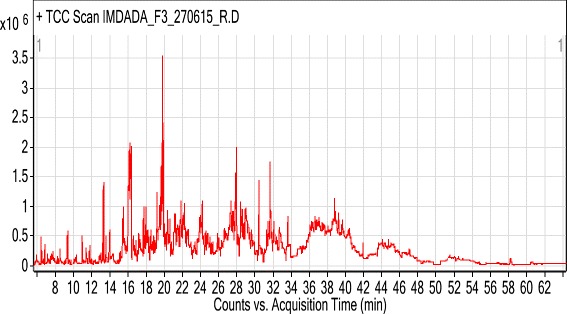
GC-MS Chromatogram of essential oil of *Rumex hastatus*

Some important components of essential oil and the chromatogram have been given in Figs. 3 and 4 respectively.

## Conclusion

Essential oil isolated for the first time from the *R. hastatus* and its chemical composition demonstrates that *R. hastatus* is a source of valuable volatile components. Based on the anticholinesterase and antioxidant results of essential oil, it can be concluded that *R. hastatus* plant may be an effective source of compounds which may lead to possible palliative therapy and cure of oxidative stresses and neurodegenerative diseases.
